# Low level of antibodies to the oral bacterium *Tannerella forsythia* predicts bladder cancers and *Treponema denticola* predicts colon and bladder cancers: A prospective cohort study

**DOI:** 10.1371/journal.pone.0272148

**Published:** 2022-08-22

**Authors:** Lise Lund Håheim, Dag S. Thelle, Kjersti S. Rønningen, Ingar Olsen, Per E. Schwarze

**Affiliations:** 1 Department of Oral Biology, University of Oslo, Oslo, Norway; 2 Institute of Basic Medical Sciences, University of Oslo, Oslo, Norway; 3 Department of Community Medicine and Public Health, University of Gothenburg, Gothenburg, Sweden; 4 Department of Paediatric Research, Oslo University Hospital, Oslo, Norway; 5 Norwegian Institute for Public Health, Oslo, Norway; University of Catania: Universita degli Studi di Catania, ITALY

## Abstract

This study explores the risk for cancer by level of antibodies to the anaerobe oral bacteria of periodontitis *Tannerella forsythia* (TF), *Porphyromonas gingivalis* (PG), and *Treponema denticola* (TD) all three collectively termed the red complex, and the facultative anaerobe bacterium *Aggregatibacter actinomycetemcomitans* (AA). The prospective cohort, the Oslo II-study from 2000, the second screening of the Oslo study of 1972/73, has been followed for 17 ½ years with regard to cancer incidence and mortality. A random sample of 697 elderly men comprised the study cohort. The antibody results measured by enzyme linked immunosorbent assay (ELISA) were used in the Cox proportional hazards analyses, and quartile risk on cancer incidence in a 17 ½ years follow-up. Among the 621 participants with no prior cancer diagnoses, 221 men developed cancer. The incidence trend was inverse, and the results are shown as 1^st^ quartile of highest value and 4^th^ as lowest of antibody levels. The results of the Cox proportional regression analyses showed that TF inversely predicts bladder cancer (n = 22) by Hazard Ratio (HR) = 1.71 (95% CI: 1.12, 2.61). TD inversely predicts colon cancer (n = 26) by HR = 1.52 (95% CI: 1.06, 2.19) and bladder cancer (n = 22) by HR = 1.60 (95% CI: 1.05, 2.43). Antibodies to two oral bacteria, *TF* and *TD*, showed an inverse risk relationship with incidence of specific cancers: *TF* bladder cancer, *TD* bladder and colon cancer. Lowered immunological response to the oral infection, periodontitis, is shown to be a risk factor in terms of cancer aetiology.

## Introduction

The levels of antibodies to particular bacteria and virus tell us about the exposure and the individual’s ability to respond to infections, and vary from person to person. Bacteraemia due to oral bacteria has been the background for exploring whether oral bacteria are a possible contributing factor involved in cancer pathogenesis. Meta-analyses have been used to summarize the prediction of periodontal disease or tooth extraction as proxy of periodontal disease on cancer risk in total or site specific forms of cancer [1–7].

Main infections that occur in the oral cavity are gingivitis, periodontitis, and caries. Untreated caries may extend to pulpitis and periapical periodontitis. Oral bacteria are members of a large consortium of microorganisms. Many of them show tissue destructing abilities depending on the oral environment as acidity related to caries and proteolysis related to periodontitis. The meta-analyses referred to above used different signs and indicators of clinical disease, varying from self-reported symptoms and tooth extraction to presence of periodontal disease or antibodies and inflammatory markers (1–8). The presence of specific oral bacteria were used in two of these analyses [1, 3] and Park et al. studied interleukin-6 and antibodies to PG [8]. Certain bacteria as PG have shown the ability of being viable but nonculturable (VBNC) bacteria [9]. They possess the ability to enter a state of low metabolic activity, but are alive when being stressed. They can return to the culturable state or resuscitate. This is anticipated to occur in the oral cavity and in distant sites. Both pathogens and non-pathogens may enter the VBNC state.

PCR and modern immunological technics allow for identification of non-cultivable bacteria and over 700 different bacteria have been identified in the oral microbiota [10]. In 1998, Socransky et al. identified and characterized clusters of oral bacteria in gingivitis and periodontitis [11]. They described the most disease-progressive bacteria in periodontitis to be three bacteria collectively termed the red complex namely *Tannerella forsythia* (TF), *Porphyromonas gingivalis* (PG), and *Treponema denticola* (TD) [12, 13]. PG is considered a keystone bacterium in the development of periodontitis and has been in focus also in cancer studies [1, 8]. Other oral bacteria as *Prevotella intermedia* (PI), *Aggregatibacter actinomycetemcomitans* (AA), and *Fusobacterium nucleatum* (FN) have also been studied with regard to cancer risk [8]. Low level of antibodies of aggressive tissue-destructing oral bacteria in individuals is feasible as a means of extra-oral spread of disease. This study investigates prospectively the antibody level to three anaerobe bacteria of the red complex TF, PG, and TD and the facultative bacterium AA on cancer incidence in a 17 ½-years prospective cohort, the Oslo II-study from 2000 [14].

## Methods

### Study population

This study include a randomized age stratified sample of 697 men from case and control groups that were available from a population-based study. Among these, 76 men had a previous cancer diagnosis. They were excluded from this study sample which finally comprised 621 men. The population-based study the Oslo II-health screening was carried out in 2000 in Oslo, Norway [14]. The aim of the initial study was to study risk and treatment of cardiovascular disease (CVD) with follow-up in men as CVD had become a major health threat among men in the 60-ies and early 70-ies in Norway. To the health survey from February 17th to June 23rd were those men invited who previously had been invited to take part in the Oslo-study 1972/73 [15]. In all, 12,764 men were invited and 6,530 attended and 5,323 men participated in both health surveys. The participants answered two questionnaires, had specific health measurements taken, and blood samples taken. One tube of EDTA/full blood and the remaining serum after planned risk factor analyses were stored at -80°C for future analyses. During the period 2004–05 analyses for antibodies to four oral bacteria (1,172) and high sensitive C-reactive protein (hs-CRP) (n = 5,323) were performed [16, 17]. The SPSS version 26 random generator program was used.

The Oslo II study protocol including description of all performed procedures were approved by the Norwegian Data Inspectorate, concession reference number 99/2282-2, included in the concession of the Oslo health study, and according to the Norwegian laws in compliance with the ethical standards of the Regional Committees for medical and health research ethics, reference no. DT ref. 03/111. The Regional Committee for Medical and Health Research Ethics approved the study protocol for Oslo II before its start in 2000. In 2003 the concession for the Oslo II study was included in the concession for the Norwegian Health Studies combined. This particular analysis is approved by the Committees for Medical and Health Research Ethics (REC South East) (reference number 2012/1837). The study complied with the 1964 Helsinki declaration and its later amendments or comparable ethical standards. A written informed consent was obtained in the 2000 health survey from all individual participants included in the study. The participants could withdraw at a later date if they so wished. The manuscript does not contain any personal identification information about the participants. The study data are not publicly available unless a permit is given by the authorities and this has not been applied for.

### Outcome

The information on cancer incidence and mortality was supplied by the Cancer Registry, Norway, and mortality by any cause was equally supplied by Statistics Norway, coordinated by the Norwegian Institute of Public Health to the study database in one linkage operation for the researcher.

### Exposure variables—antibody analyses

The ELISA antibody analyses have been previously described [16]. The serum was analysed for the three anaerobe bacteria TF, PG, and TD and the facultative anaerobe AA at the Norwegian Institute for Public Health according to standard methodology using the bacteria cultivated at the Institute of Oral Biology, Dental Faculty, University of Oslo. A homogenous suspension of bacteria with a protein concentration of 5 μg/ml was coated onto the ELISA plates (all at one time) to react with the antibodies of the serum samples. After overnight incubation, the plates were stored for up to 14 days at 4°C. Plates were washed and samples or serum calibrator (used as an internal standard) added, and the plates were incubated for 2 hours at room temperature. After another washing a conjugated secondary antibody (Polyclonal Rabbit Anti Human IgG/AP, D0336, 1: 1,000) was then added and the plates were incubated at room temperature for another 2 hours. Thereafter the plates were washed again and the substrate for colour development was added. The colour development was registered by optical density (OD) and the result was recorded and calculated as a percentage of the serum calibrator. A series of dilutions were used to validate the concentrations. Sample dilution curves were compared for deviation from parallelism. Mean values were used unless large deviations were observed and then median values were chosen.

### Statistical methods

Descriptive measurements of the participants are presented by mean with standard deviation (SD) or number and percent, including the bacterial values per cancer diagnosis. The optical ELISA antibody readings were given by mean, median, minimum and maximum values, and quartile values of all included (n = 697), and mean, median and standard deviation per relevant cancer diagnoses. The quartiles values were used on an ordinal scale in the statistical analyses. The 10th version of International Classification of Diseases (ICD-10) codes were available for cancer incidence and mortality and the categories used followed the ICD-10 anatomical site groups. Survival analyses were done by Cox proportional hazards regression analyses in univariate and multivariate analyses. The outcome was incidence of cancer. Person time was calculated from day of screening in 2000 to date of first registered cancer diagnoses as incidence or death. Cases registered were censored at day of death for other causes. Last day of follow-up was 31.12.2017.Univariate analyses were age adjusted and multivariate analyses adjusted for age, daily smoking and education, known confounders for oral infections and cancer. Age and education were used on a continuous scale and daily smoking dichotomized as yes (daily smoking) or no (never and past smoker). Colon cancer was selected for subgroup analyses. Interaction analyses between daily smoking and each bacterial antibody quartile status were performed for bladder cancer as daily smoking is well established as a risk factor. Kaplan-Meier plots of the cumulative survival of total cancer incidence were made per quartile levels of each of the four bacteria. Histograms were made to illustrate incident cancers per quartile antibody level of the four bacteria for selected outcomes. A P-value of < 0.05 was adhered to and the statistical package SPSS 26 was used.

## Results

### Description of participants

Available for analyses were data on a random sample of 697 elderly men of whom 76 had a prior diagnosis of cancer hence results of the follow-up for 621 men are presented (Table 1). Basic characteristics reported are age, education, daily smoking, Body Mass Index (BMI), high sensitivity-C-reactive protein (hs-CRP), and alcohol consumption. Men with cancer were older (71.3 years) than non-cases (69.9 years) and had longer education (12.9 years versus 11.9 years). No significant differences were observed for the self-reported medical conditions asthma, diabetes, and bronchitis/emphysema, nor for differences in health service use such as visits to the general practitioner and the dentist the last 12 months. The men had previously taken part in the first health survey in the Oslo study in 1972/73 and many risk factors had changed in these men.

**Table 1:** Baseline characteristics, self-reported medical history, and health service use for incident cases of cancer and no cancer of a random sample of participants from the Oslo II-study in 2000.

Covariates	Cancer incidence 2000–2017		
	Cancer	No cancer	P-value
	n = 113	n = 508	
*Basic characteristics*			
Age, year, mean SD	71.3 (3.9)	69.9 (5.5)	**0.014**
Education, year, mean SD	12.9 (4.0)	11.9 (3.7)	**0.008**
Daily smoking, n (%)	19 (16.8%)	113 (22.2%)	0.252
BMI, kg/m^2^, mean SD	26.4 (3.2)	26.4 (3.4%)	0.893
Hs-CRP, mean SD	4.1 (12.2)	3.4 (7.7)	0.857

Excluded are men reporting prior history of cancer. Results of p<0.05 are in bold.

### Cancer diagnoses

**Fig 1:**
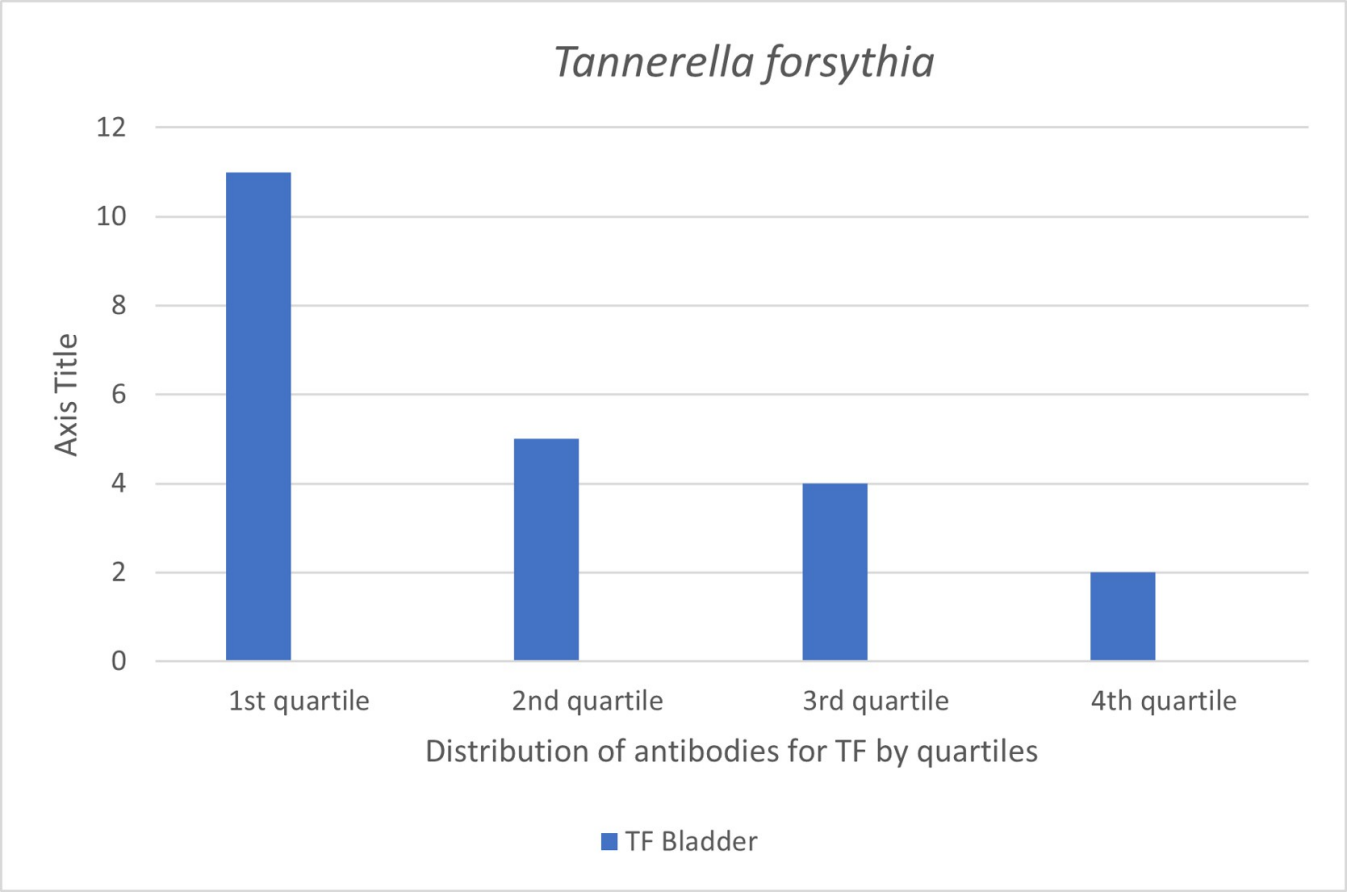
Number of incident cancer cases of bladder by quartile antibody levels of *Tannerella forsythia* (TF). Quartile (Q) values are Q1 9–35, Q2 36–58, Q3 59–104, Q4 105–1204.

**Fig 2:**
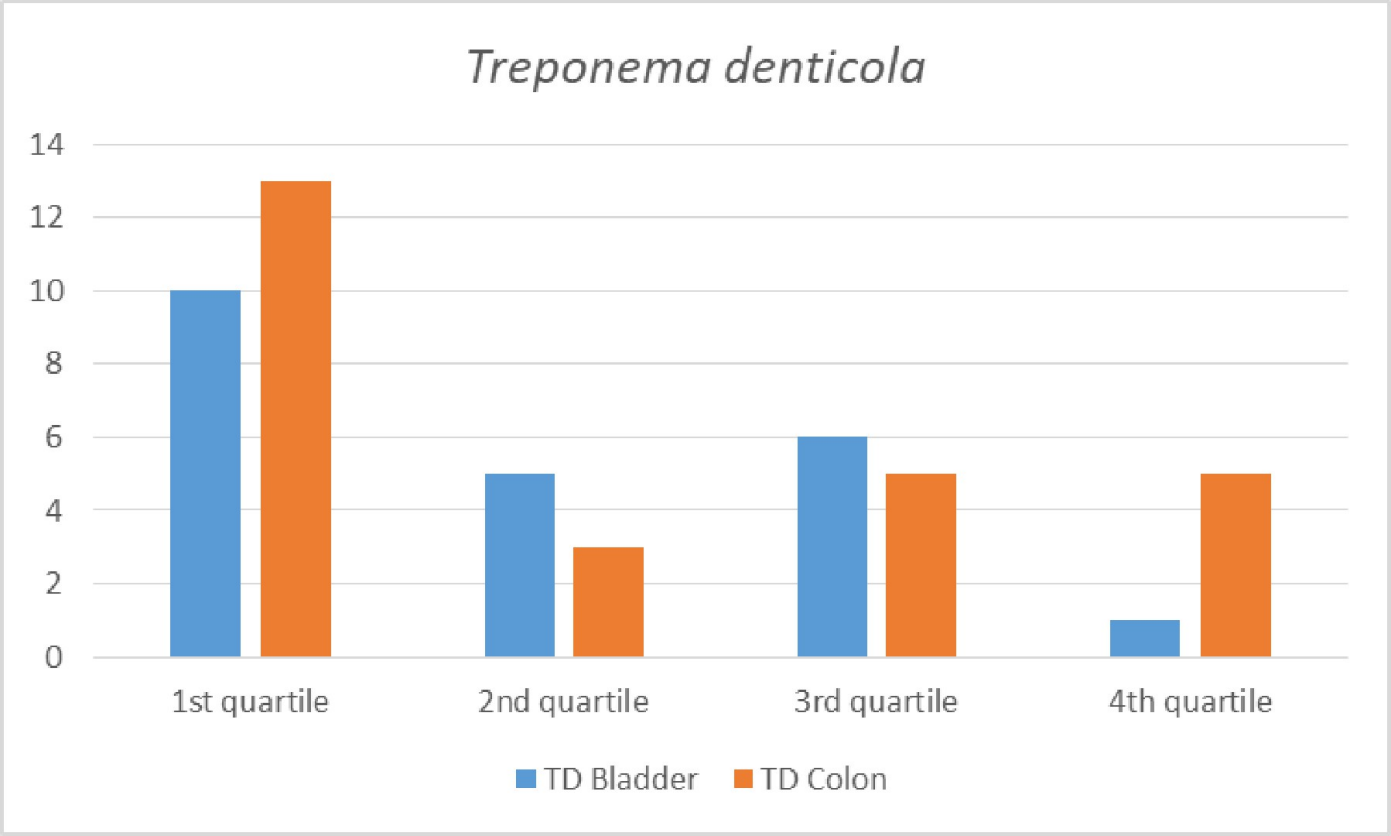
Number of incident cancer cases of bladder and colon by quartile antibody levels of *Treponema denticola (TD)*. Quartile (Q) values are Q1 1–28, Q2 29–44, Q3 45–73, Q4 74–1240.

**Fig 3:**
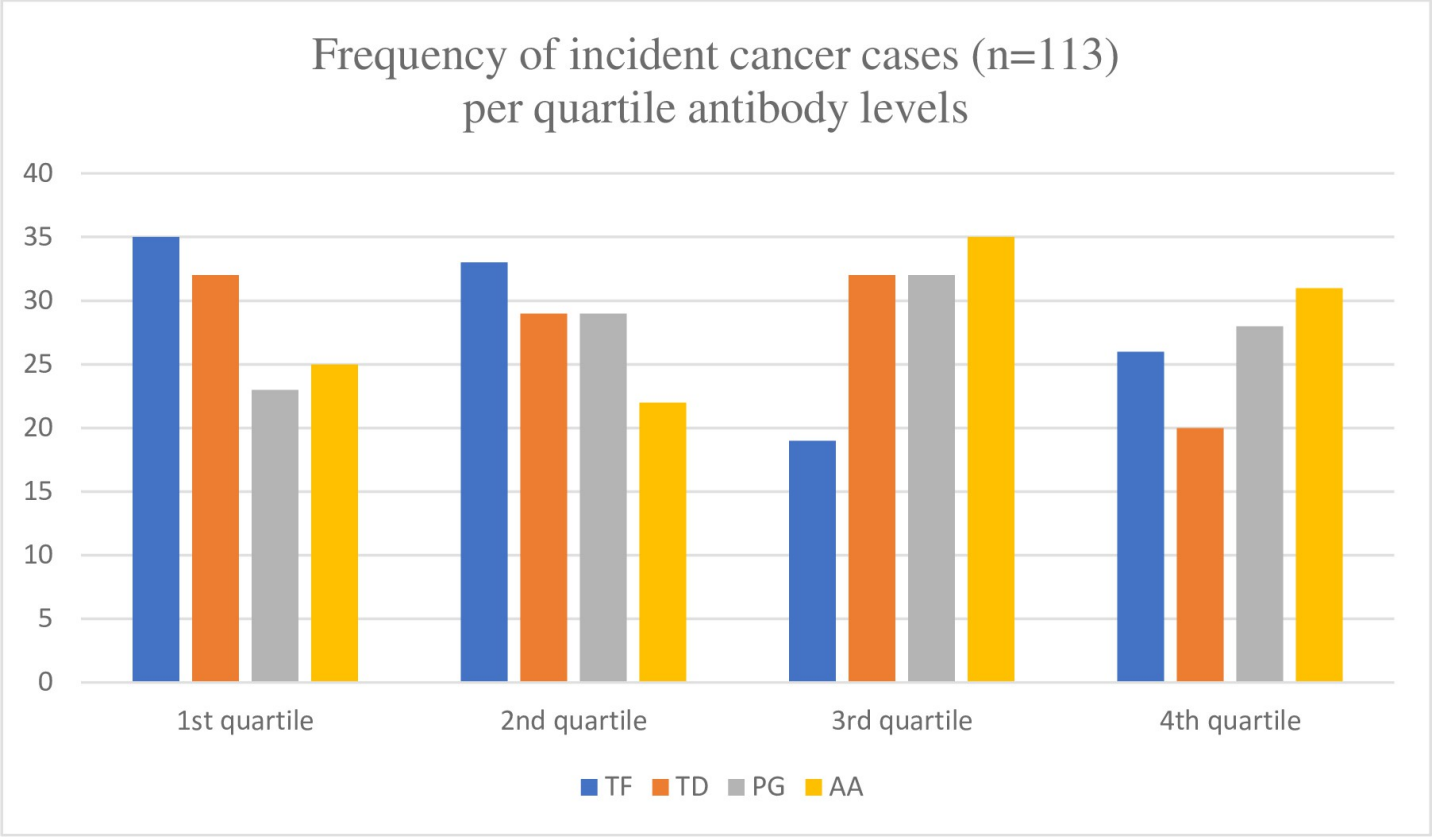
Frequency of incident cancer cases (n = 113) per quartile antibody levels of the oral bacteria *Tannerella forsythia* (TF), *Porphyromonas gingivalis* (PG), *Treponema denticola* (TD) and *Aggregatibacter actinomycetemcomitans* (AA). Quartile (Q) levels: TF Q1 1–28, Q2 29–44, Q3 45–73, Q4 74–1240, TD Q1 1–28, Q2 29–44, Q3 45–73, Q4 74–1240, PG Q1 1–28, Q2 29–44, Q3 45–73, Q4 74–1240., AA Q1 1–28, Q2 29–44, Q3 45–73, Q4 74–1240.

**Fig 4:**
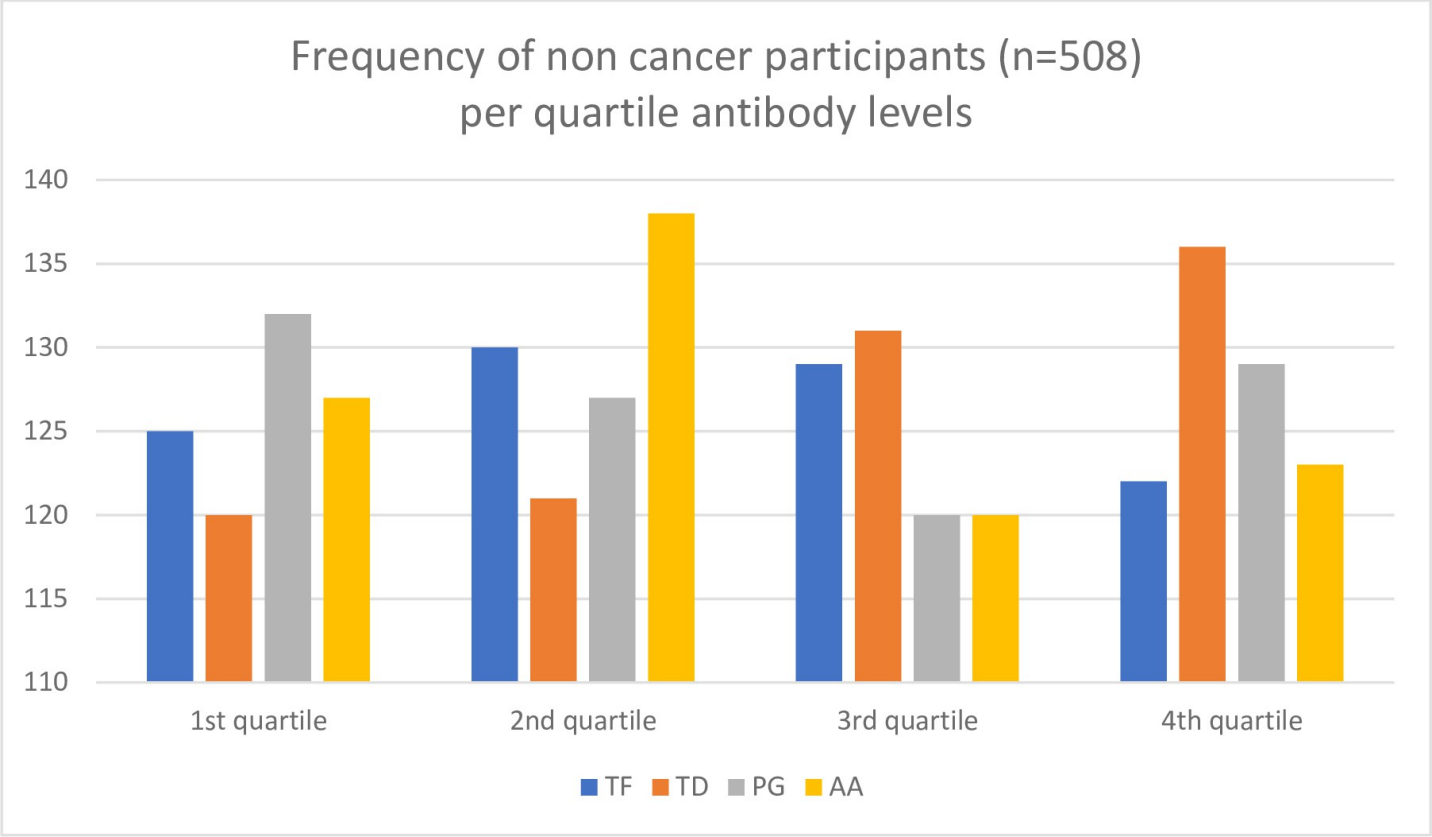
Frequency of non-cancer participants (n = 508) per quartile antibody levels of the oral bacteria *Tannerella forsythia* (TF), *Porphyromonas gingivalis* (PG), *Treponema denticola* (TD), and *Aggregatibacter actinomycetemcomitans* (AA). Quartile (Q) levels: TF Q1 1–28, Q2 29–44, Q3 45–73, Q4 74–1240, TD Q1 1–28, Q2 29–44, Q3 45–73, Q4 74–1240, PG Q1 1–28, Q2 29–44, Q3 45–73, Q4 74–1240., AA Q1 1–28, Q2 29–44, Q3 45–73, Q4 74–1240.

Our approach was to stratify the participants by their ICD-10 cancer diagnoses categories (Table 2). The participants were men and 12 categories were applicable. There were too few cases in a number of these categories to enable meaningful analyses as for example the group of lip, oral cavity, and pharynx cancer that counted only five cases. As oral bacteria are in focus, this is regrettable but it shows that cancers of oral and pharyngeal origin are rare forms of cancer. Categories considered to have sufficient number of cases for analyses were digestive organs (mainly colon and rectum cases) (n = 56), lip, oral cavity, pharynx, bronchus, and lungs (n = 23), malignant melanoma and neoplasms of skin (n = 27), male genital organs (n = 63), bladder (n = 22), and malignant neoplasms of lymphatic, haematopoietic and related tissue (n = 19). All cases prior to 2000 were excluded. These groups of cases were used in the Cox proportional hazards regression analyses. The distribution of cases per quartiles of antibodies of the oral bacteria TF per and bladder cancers and TD per bladder and colon cancers are shown in Figs 1 and 2. For both TF and TD the skewed distribution of 1st quartile having the highest number of cases per diagnosis is obvious, but more so with TF (Figs 1 and 2). Case distribution of all four bacteria for total cancer incidence show the inverse relationship with TF cases but not for non-cases are shown in Figs 3 and 4.

**Table 2:** Descriptives of the ELISA readings of the antibodies to the bacteria *Tannerella forsythia*, *Treponema denticola*, and *Porphyromonas gingivalis*, collectively termed the red complex, and *Aggregatibacter actinomycetemcomitans*: The Oslo II-study health screening in 2000.

Cancer diagnoses*	Antibodies of serum ELISA readings			
	mean, median, standard deviation			
	*Tannerella forsythia*	*Treponema denticola*	*Porphyromonas gingivalis*	*Aggregatibacter actinomycetem-comitans*
**Overall values**				
n = 697				
Mean, median	96.9, 58.0	67.0, 44.0	215.8, 88.5	102.2, 55.0
Standard deviation	122.1	89.3	413.4	181.3
Minimum	9	1	9	7
25% percentile	35	28	52	32
50% percentile	58	44	89	55
75% percentile	104	73	195	104
Maximum	1204	1240	5299	3042
**Cancer diagnoses**				
selected ICD-10* codes				
n = Incident cases 2000–2017				
Lips, oral cavity, pharynx, bronchus, lungs, C00-C14, C30-C39, n = 23	119.26, 43.00 228.79	68.13, 48.00 69.33	225.39, 123.00 361.61	148.00, 77.00 300.39
Digestive organs, C15-C26, n = 56	100.63, 49.00 134.74	62.07, 38.50 70.22	263.11, 90.50 598.59	108.52, 50.00 116.12
- Colon, C18, n = 26	99.96, 43.00 148.63	55.42, 29.50 63.95	207.04, 105.00 219.75	98.19, 47.50 100.10
Bladder, C64-C68, n = 22	54.55, 35.50 45.04	48.91, 37.00 66.73	280.86, 180.00 337.56	97.82, 80.50 96.32
Malignant melanoma and neoplasms of skin, C43-C44, n = 27	132.41, 57.00 246.39	99.22, 56.00 214.50	344.19, 64.00 786.96	146.22, 62.00 291.33
Male genital organs, C60-C63, n = 63	91.90, 46.50 120.89	69.81, 47.00 72.55	122.27, 85.00 123.61	99.08, 58.00 111.76
Lymphatic, haematopoietic and related tissue, C81-C96, n = 19	121.11, 58.00 164.93	43.00, 42.00 23.31	146.39, 71.00 244.81	57.95, 53.00 35.67

* ICD-10 codes = International Classification of Diseases Version 10

### ELISA antibody readings

The ELISA optical readings are described by mean, standard deviation (SD), and minimum and maximum values. Quartile values illustrate some differences in Table 2. Highest maximum levels ranged from 1,204 of TF to 5,299 of PG. Lowest level ranged from 1 (TD) to 9 (PG and TF). The standard deviations were large and quartile values were found to be suitable to use for further analyses. Equivalent values per relevant cancer diagnosis are presented in Table 2. The highest mean, median and standard deviation values were recorded for PG overall and per selected cancer diagnoses. The distribution of cases comparing all cancer cases (n = 113) per quartile of bacterial antibody ELISA values are shown in Fig 3 and equivalent presentation for non-cases (n = 508) in Fig 4.

### Survival analyses

**Fig 5:**
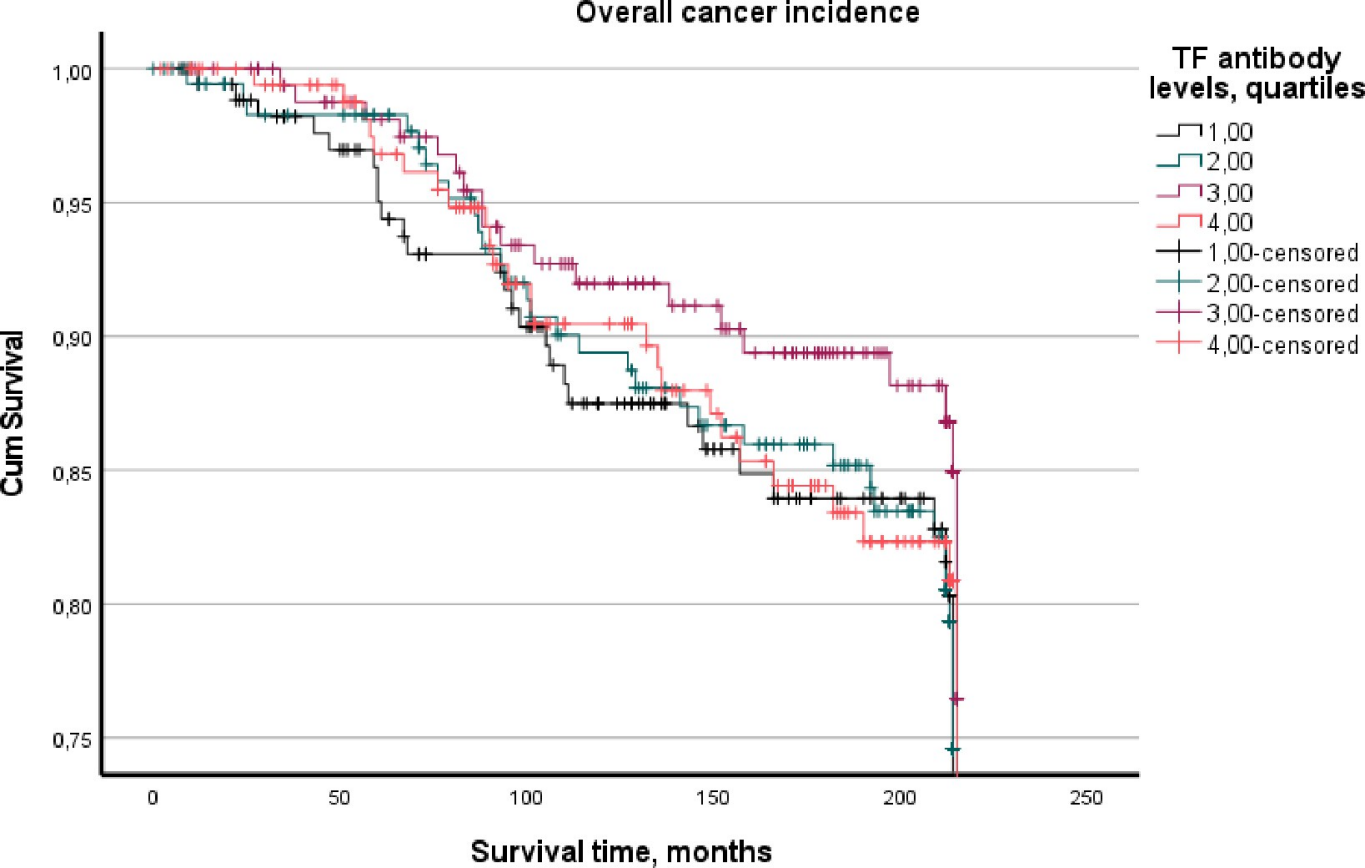
Overall cumulative cancer incidence stratified on quartiles of *Tannerella forsythia* antibody levels.

**Fig 6:**
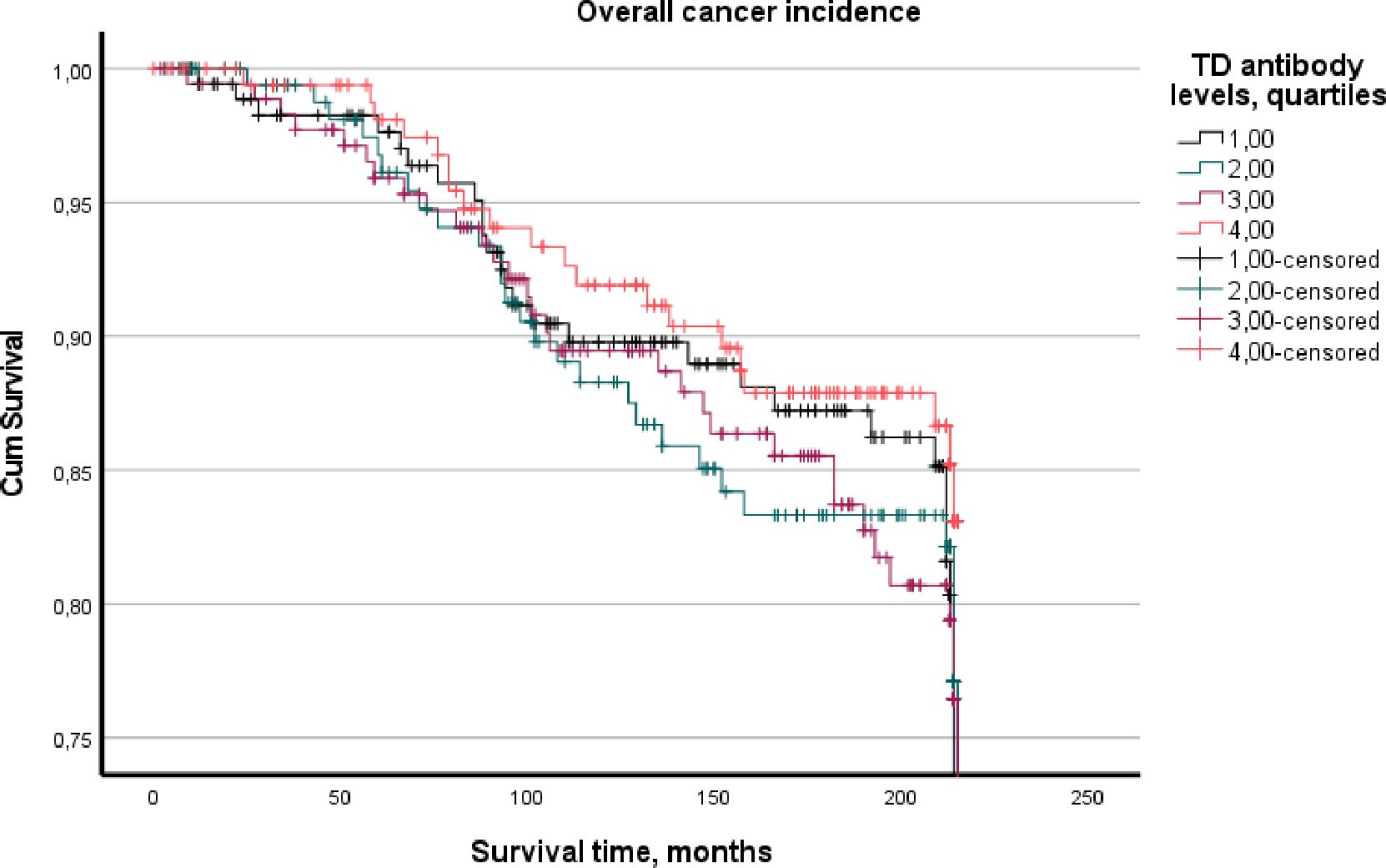
Overall cumulative cancer incidence stratified on quartiles of *Treponema denticola* antibody levels.

The survival analyses by the Cox method of proportional regression analyses was done by age, age and daily smoking, and age, daily smoking and education adjusted models (Table 3). Results are given for the chosen cancer categories by Hazard ratio (HR), and 95% confidence interval (CI). The preliminary analyses showed that there was an increased risk by low levels of antibody levels analysed by quartile (Q) values. In presenting the results of this inverse relationship, Q1 was given the level 4, Q2 the level 3 and so forth to get a better presentation of the risk level of the estimates. Three significant findings by quartile antibody levels were observed. First, low levels of both TF and TD antibodies increased the risk for bladder cancer, for TF by a HR = 1.71, 95% CI: 1.12, 2.61 and for TD by a HR = 1.61, 95% CI: 1.05, 2.44. TD predicted colon cancer by HR = 1.52, 95% CI: 1.06, 2.19. Other sites of the digestive system or overall gastrointestinal cancers were not significant. Smoking is an established risk factor for bladder cancer and promotes periodontitis. Consequently, we performed an interaction analysis of each bacterium and daily smoking on bladder cancer but no interaction was observed. The significant levels of the interaction terms were for TF 0.521, TD 0.516, PG 0.636, and AA 0.602. The Kaplan-Meier plots of the overall cancer incidence assessment of TF and TD showed differences between the bacteria (Figs 5 and 6). The 1st quartile of TF showed lowest cumulative survival for TF through most of the follow-up period. Plots per diagnosis were not made as there were few cases in some of the quartiles.

**Table 3:** Age, daily smoking and years of education adjusted multivariate Cox proportional regression analyses of antibodies to four oral bacteria on cancer incidence.

Cancer diagnoses*	Antibodies by quartiles 1–4 inversely; 1^st^ quartile has value 4 HR and 95% confidence interval			
	*Tannerella forsythia*	*Treponema denticola*	*Porphyromonas gingivalis*	*Aggregatibacter actinomycetem-comitans*
Lips, oral cavity, pharynx, bronchus, lungs, n = 23	1.16	0.95	0.85	0.35
	0.79, 1.71	0.65, 1.39	0.58, 1.22	0.56, 1.22
Digestive organs, C15-C26, n = 56	1.07	1.27	0.92	0.94
	0.84, 1.36	0.99, 1.61	0.73, 1.16	0.74, 1.21
- Colon, n = 26	1.27	**1.52**	0.87	1.06
	0.89, 1.83	**1.06, 2.19**	0.62, 1.23	0.74, 1.53
Malignant melanoma and neoplasms of skin, n = 27	1.10	0.85	1.02	0.89
	0.78, 1.54	0.60, 1.20	0.73, 1.42	0.62, 1.26
Male genital organs, n = 63	1.16	0.96	1.07	0.90
	0.93, 1.46	0.76, 1.20	0.86, 1.34	0.71, 1.13
Bladder, n = 22	**1.71**	**1.60**	0.74	0.94
	**1.12, 2.61**	**1.05, 2.44**	0.50, 1.09	0.64, 1.38
Lymphatic, haematopoietic and related tissue, n = 19	1.01	1.25	1.32	1.25
	0.67, 1.52	0.82, 1.88	0.86, 2.03	0.81, 1.92

* n = Incident cases 2000–2017 Analyses adjusted for known confounders age, daily smoking, and years of education

The risk by antibody levels was inverse. To present this increased risk by low levels, the reference group was changed from quartile 1 to quartile 4. Cancer prior to 2000 have been excluded. Results of p<0.05 are in bold.

## Discussion

Antibody measurements of oral bacteria reflect past infection and to what extent an individual’s immune system has responded. This study examined the hypothesis that the antibody level is a predictor for cancer incidence and we found that those with the lowest levels of antibodies for the two oral bacteria TF and TD had a higher risk of bladder cancer. Low levels of TD were also associated with increased risk of colon cancer. We have reported earlier that low level of the antibody to the oral bacterium TF predicted fatal cardiovascular disease in individuals with a history of myocardial infarction [18].

Other studies have used different exposures as presence and degree of periodontal disease, gingivitis, tooth extractions, or caries depending on the research focus. The meta-analyses mentioned used presence of periodontal disease, antibodies to oral bacteria, edentulism, and tooth loss mainly on different cancer diagnoses [1–8]. Michaud et al. concluded in their meta-analyses of 46 out of 217 identified publications in the years 2011–2016 that there was increased risk for cancer by periodontal disease or tooth loss when used as a surrogate for periodontal disease [2]. Periodontal disease increased the risk of total cancer by 14–20% in five studies controlled for smoking. They found an increased risk for lung cancer, RR = 1.33, 95% CI: 1.19, 1.49 [2]. Maisonneave et al. found relative risk (RR) estimates from eight studies of periodontal disease of RR = 1.74, 95% CI: 1.41, 2.15 and edentulism RR = 1.54, 95% CI: 1.16, 2.05 for pancreatic cancer [19]. Michaud et al. also studied pancreas cancer and antibodies to oral bacteria in a case control study within the large European cohort study (EPIC) and studied antibody levels of 25 oral bacteria and pancreatic cancer [20]. They found OR = 2.14 (95% CI 1.05 to 4.36) for high versus low level of PG. They also observed that a cluster of higher levels of antibodies had a 45% lower risk of pancreatic cancer than lower levels, OR = 0.55 (95% CI 0.36, 0.83). In another case control study on oesophagal squamous cell carcinoma (ESGG), 96 cases with ESCC, 50 cases with esophagitis, and 80 controls had preoperative serum immunoglobulin G and A antibodies measured [21]. It was observed that the median serum levels of IgG and IgA for PG were significantly higher in ESCC patients than non-ESCC controls. Bracci et al. concluded from epidemiological studies that the risk for pancreatic cancer by periodontal disease ranged from 1.5 to 2.0 with RR of meta-analysis equal to 1.74 and observed that oral bacteria in prediagnostic blood may be related to pancreas cancer [3]. Xiao et al. examined the risk of cancer incidence by periodontal bacterial infection and found TD and *P*. *intermedia* to be significant predictors but not TF, *PG*, *F*. *nucleatum* (FN) or AA [7]. Periodontal bacterial infection reduced survival of cancer. PG and FN were studied by Park et al. as potential biomarkers for cancer and IL-6, an inflammatory marker for oral squamous cell carcinoma (OSCC) in 62 cases and 46 control persons [8]. Elevated levels of PG and Interleukin 6 (IL-6) were significantly associated with OSCC and a high serum level of IL-6 also predicted poor survival [8]. In all, the results vary but several studies show an association between oral health and oral microbiome and cancer from different populations in different countries of different study design and size. Other relevant factors have been studied. A common associated factor between periodontitis and cancer is elevated level of hs-CRP [6]. Treatment of periodontal disease has shown a reduction in the hs-CRP level [22]. Smoking can lead to DNA-methylation of genes that normally are tumour suppressants resulting in their inactivation with regard to protecting cells against cancer development [23]. Genetic analyses have included analyses of p53 arginine mutations detected in patients with pancreatic cancer [23]. PG, TF, TD, and *P*. *intermedia*, possess the peptidyl arginine deaminase (PAD) enzymes. The Pro allele p53Arg72-Pro is a risk factor for the development of pancreatic cancer and the author hypothesize that there is a genetic explanation to these anaerobic oral bacteria known for their tissue destruction capabilities that contribute in pancreatic cancer development.

It is difficult at this stage, to ascertain which are the microbiological or immunological mechanisms that explain the observed association between the low level of certain oral antibodies to specific cancer diagnoses. All the participants of the study had some antibodies, however, little for some of the men, indicating exposure to chronic periodontal infection. This study indicate that there is a certain IgG deficiency due to low production of immunoglobulins G(IgG), hence these men were prone to get infections. This impairment of the immune system can be due to genetic mutations, certain diseases, certain drugs and possibly some environmental exposures. We hypothesize that the presence of oral bacteria in the circulation cause changes and immunologic reactions also distant to the initial oral infection due to the observed deficient immunologic response.

Low level of antibodies permits spread of disease. The three bacteria in the red complex benefit from each other’s microbiological properties in order to survive under anaerobe conditions. It is conceivable that the bacteria are part of a more general immunological mechanism connected to bacterial infections or are associated with ongoing bacterial infections. Smoking is an established risk factor for bladder cancer [24]. We can only speculate that our results on bladder and colon cancers are influenced by the late effect of daily smoking on the immunological system. Antibody levels to these oral bacteria are independent of daily smoking status for these outcomes in our explorative analyses. Smoking (and other factors), however, is known to lower the immune response and may therefore contribute to the carcinogenesis. We hypothesize that oral bacteria when present in the circulation may exacerbate already ongoing cancer disease processes. One observation in support is that haemorrhage is a presenting symptom; haematuria of bladder cancer and rectal bleeding of colon cancer [25, 26]. Another approach could be to investigate how deficient IgM antibody production to bacteria is related to cancer immune types. Thorsson et al. performed immunogenomic analyses of over 10,000 tumours of 33 different cancer types and identified six different immunologic subtypes [27]. These were termed wound healing, IFN-g dominant, inflammatory, lymphocyte depleted, immunologically quiet, and TGF-b dominant and the groups were distinguished by a number of different parameters on cellular and genomic levels. Isola et al. have studied how the nod-like receptor family pyrin domain-containing protein-3 (NLRP3) complex inflammasome potentially play an important role in the development of periodontitis and diabetes [28]. Periodontitis activates the NLRP3 inflammasome in serum and saliva. Another approach was taken by Ferlazzo et al. who studied whether genetic polymorphisms of the (MTHFR) enzyme may influence DNA methylation [29]. The authors concluded that hypermethylation of cancer-related genes may be affected by *MTHFR* polymorphisms in the development of Oral Squamous Cell Cancer (OSCC). Both studies explore the direct effect in the oral cavity on cancer development. A further understanding of these relationships may identify which types of tumours that are related to bacterial immunity and guide prophylactic measures and treatment.

Our study design was a random sample of elderly men selected within a well-established cohort. The screening was performed in 2000 and a long prospective follow-up of the antibody measurements was planned. From the results of the screening in 2000, we know that health variables like daily smoking, BMI, and systolic blood pressure have changed over 28 years since the first screening in 1972/73 [14, 15]. Noticeable was the reduction in daily smokers. The study sample size was reasonable in order to provide results distinguishing between cancer diagnoses in these elderly men. The ELISA methodology provided results on a continuous scale that was desired for epidemiological purposes to study risk distribution within and between antibody levels of the four bacteria. The large standard deviations made presentation by quartiles preferable. The quartiles also permitted quartile comparisons and trend observations rather than using point estimates for statistical purposes. The choice of bacteria was based on prior knowledge of bacteria involved in periodontitis, the most severe and tissue destructing bacteria resident in the soft tissues and in close approximation to alveolar bone gave the opportunity and ability for external spread to and by the circulation. This study does not give a direct pathophysiologic explanation as to how these bacteria are involved in cancer pathology. The spread by bacteria and possibly bacterial products describe a mode of action. The bacteria found to be predictive of cancer in this study, TF and TD, are tissue destructing and part of the red complex of bacteria that are present together in periodontitis and they benefit from each other in advancing disease [12, 30].

A limitation is gender as the study includes men only. Age is another limitation of this study although the incidence of cancer, in general, increases with age. Age is an underlying factor to be taken into account in understanding the results as risk factors of cancer generally have a long latency period. With regard to oral infections, they are frequently chronic in nature with acute exacerbations in prone individuals. In searching for associations in data, it is common to perform many tests of significance. One common approach is to correct for multiple tests by the Bonferroni method. This yields a low p-value and fewer associations discovered in order to reduce the possibility of findings being incorrect by chance. However, in explorative analyses one does not know in advance which form of cancer is significant or not. Rothman argues that researchers loose information of associations of importance in clinical research when using the restricting Bonferroni method rather than using a fixed p-value [31].

In conclusion, our 17 ½ year follow-up study provides evidence towards an association to two different cancer diagnoses, colon and bladder cancer, by low levels of antibodies to the oral bacteria TF and TD, both members of the red complex bacteria in periodontitis. The low antibody level risk infer a pathologic mode of infection spread of oral bacteria. We suggest that optimal oral hygiene may reduce the risk observed, and further research is needed to explore the risk by immunologic factors related to the oral microbiome on cancer incidence and its pathophysiology in other populations to explore the generalizability of our results.
